
JOURNAL INFORMATION
==============================
NLM Title Abbreviation: ScientificWorldJournal
Journal ID: ScientificWorldJournal
NLM Journal ID: 101131163
Journal ID: TSWJ

The Scientific World Journal

ISSN: 2356-6140
EISSN: 1537-744X
Publisher: Wiley

ARTICLE INFORMATION
==============================
PMCID: PMC5848851
PMID: 19030758
DOI: 10.1100/tsw.2008.139
Article ID: 517357
Article version: 1
Subjects: Clinical Image

Reversal of Bilateral Rosette Cataracts with Glycemic Control

Ramkumar Hema
Basti Surendra *
Department of Ophthalmology, Northwestern University Feinberg School of Medicine, Chicago, USA
*Surendra Basti: sbasti@northwestern.eduAcademic Editor: Daniel Batlle

Publication date: 2008
Electronic publication date: 2008 Nov 19
Volume: 8
First page: 1150
Last page: 1151
Received 2008 Sep 11; Accepted 2008 Oct 5
Copyright: Copyright © 2008 Hema L Ramkumar and Surendra Basti.
Copyright year: 2008
License: This is an open access article distributed under the Creative Commons Attribution License, which permits unrestricted use, distribution, and reproduction in any medium, provided the original work is properly cited.
License URL: https://creativecommons.org/licenses/by/3.0/

Keywords: diabetic cataracts, Rosette cataract, hyperglycemic cataract, reversible cataract

Funding: Research to Prevent Blindness

==============================
Clinical Image
*TheScientificWorldJOURNAL* (2008) 8, 1150–1151
ISSN 1537-744X; DOI 10.1100/tsw.2008.139

TheScientificWorldJOURNAL
www.thescientificworld.com

### Reversal of Bilateral Rosette Cataracts with Glycemic Control

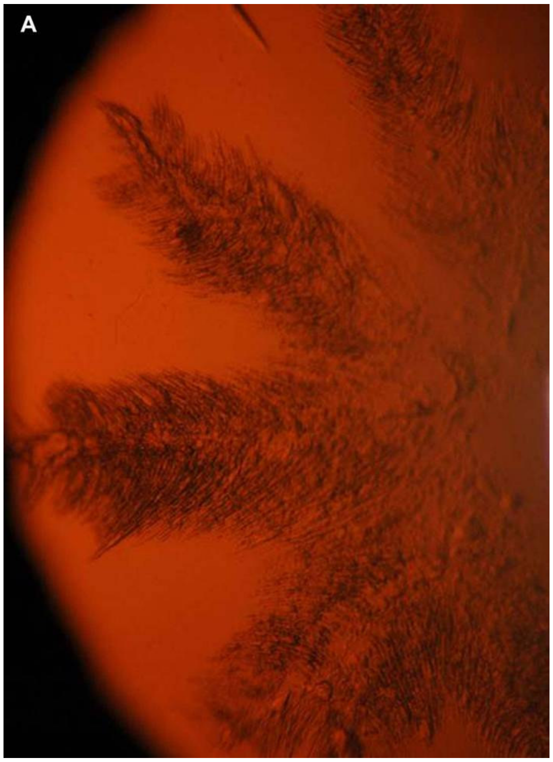

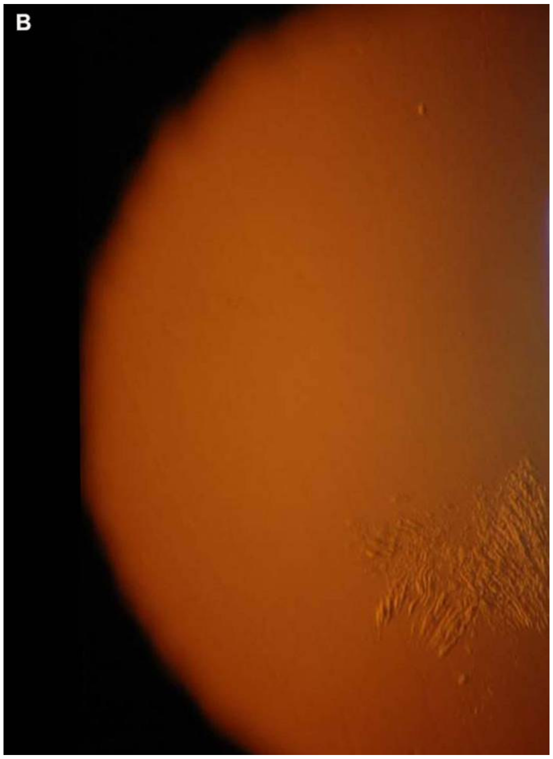

Hema Ramkumar and Surendra Basti*

*Department of Ophthalmology, Northwestern University Feinberg School of Medicine, Chicago*

**E-mail**: hema@md.northwestern.edu**;** sbasti@northwestern.edu

*Received September 11, 2008; Accepted October 5, 2008; Published November 19, 2008*

**KEYWORDS**: diabetic cataracts, Rosette cataract, hyperglycemic cataract, reversible cataract

A 58-year-old woman diagnosed with type-2 diabetes mellitus 3 weeks prior to admission presents to the emergency room with a chief complaint of decreased vision for the past 2 weeks and polyuria. She reports blood sugars in the 400s for the preceding 3 weeks. Her blood glucose on admission was 500 and her HbA1c was 15.5%. Her ophthalmic exam was positive only for a visual acuity of 20/400 and feather-

*Corresponding author.
©2008 with author.
Published by TheScientificWorld; www.thescientificworld.com shaped posterior subcapsular lens opacities (Fig. A) bilaterally. Glycemic control was quickly achieved with insulin and she was discharged. At the 2-week follow-up visit, her vision was 20/60 and the cataract had predominantly reversed (Fig. B). One month later, the lens was clear and her vision was 20/30. Two months later, her vision was 20/20. She reports maintaining blood sugars in the 100s since discharge.

The lens equatorial epithelium migrates first to the posterior subcapsular cortex and is affected most by hyperglycemia. Hypoinsulinemia causes sorbitol accumulation in the lens and fluid influx[1]. Cellular hydration delineates lens fibers, which are normally feather shaped, and impairs vision. Administration of insulin and fluids causes conversion of sorbitol to fructose, which diffuses out[2]. Reduced lens osmolarity allows vacuole resorption, cataract reversal, and vision improvement.

Acute hyperglycemia-induced cataracts are reversible with prompt glycemic control[3]. Uncorrected, hyperglycemia causes permanent cataracts that must be managed surgically. With acute worsening of vision due to cataract formation in the setting of hyperglycemia, supportive therapy with glycemic control and observation alone can provide a dramatic improvement in vision in a few weeks.

### ACKNOWLEDGMENTS

Dr. Basti's work is supported in part by a grant from Research to Prevent Blindness, New York to the Department of Ophthalmology, Northwestern University Feinberg School of Medicine.

### REFERENCES

1. Powers, A.C. (2007) Diabetes mellitus: acute complications of DM. In *Harrison's Principles of Internal Medicine*. 16th ed. McGraw-Hill, New York.
2. Tasman, W. (1992) The lens: biochemistry, glucose metabolism. In *Duane's Foundations of Clinical Opthalmology*. Vol. 2. Lippincott Williams & Wilkins, Philadelphia. Chap. 10.
3. Epstein, D.L. (1976) Reversible unilateral lens opacities in a diabetic patient. *Arch. Ophthalmol.* **94,** 461–463.

**This article should be cited as follows:**

Ramkumar, H. and Basti, S. (2008) Reversal of bilateral rosette cataracts with glycemic control. *TheScientificWorldJOURNAL* **8**, 1150–1151. DOI 10.1100/tsw.2008.139.
